# Accurate Broadband Permeability Measurement of Thin Magnetic Films in a Short-Circuited Microstrip Fixture

**DOI:** 10.3390/ma19112294

**Published:** 2026-05-28

**Authors:** Pavel A. Ivanov, Dmitry A. Petrov, Polina A. Zezylina, Ilya V. Komarov, Alexey V. Osipov, Sergey S. Maklakov, Konstantin N. Rozanov

**Affiliations:** Institute for Theoretical and Applied Electromagnetics, Russian Academy of Sciences, Moscow 125412, Russia; dpetrov-itae@yandex.ru (D.A.P.); zez-p@yandex.ru (P.A.Z.); komarov@itae.ru (I.V.K.); avosipov@mail.ru (A.V.O.); squirrel498@gmail.com (S.S.M.);

**Keywords:** thin magnetic films, complex permeability, broadband characterization, microstrip, offset-short calibration, microwave measurements

## Abstract

A broadband method for measuring the complex permeability of thin magnetic films using a short-circuited microstrip fixture is presented. The method is based on full one-port offset-short calibration implemented directly in the fixture with a movable shorting wall, reducing sensitivity to coaxial-to-strip transition imperfections and eliminating the need for its precise optimization. Field-dependent normalization to the empty fixture reduces systematic errors from the external magnetic field, and a correction for residual saturated permeability improves retrieval accuracy. The method was validated on Co, supermalloy, and FeCo films on flexible PET substrates. Retrieved spectra agreed well with reference coaxial data and with the spectrum reconstructed from static magnetic measurements. In the present implementation, broadband spectra were obtained from 0.1 to 20 GHz with no significant systematic distortion, indicating that the proposed approach is suitable for accurate broadband characterization of thin magnetic films without reference standards or precise optimization of the coaxial-to-strip transition.

## 1. Introduction

Broadband characterization of complex permeability of magnetic thin films is important for both materials research and microwave engineering. Magnetic thin films are used in high-frequency components such as inductors, transformers, filters, electromagnetic shields, and noise suppressors, and their performance is governed not only by the resonance frequency, but by the broadband frequency dependence of the real and imaginary parts of permeability. Depending on the target application, a high μ′ with low μ″ may be required, or, conversely, enhanced magnetic loss in a specific frequency range. Therefore, broadband measurement of the complex permeability spectrum is essential for both understanding magnetization dynamics and optimizing thin-film materials for practical RF and microwave devices [1].

Microwave measurement approaches for thin magnetic films may be generally divided into FMR spectrometers and RF permeameters [2,3]. Although both techniques are used for studying magnetization dynamics and may employ similar fixture structures, their measurement objectives are different. FMR spectrometers are primarily intended to characterize the resonance response and to extract quantities such as resonance frequency and linewidth, whereas RF permeameters are designed to determine the absolute complex permeability over the frequency range relevant to the intended application of the material. Accordingly, RF permeability measurements require broad measurement bandwidth, accurate calibration, and high sensitivity to weak magnetic signals.

Within RF permeameters, thin-film measurement methods may be generally divided into pickup-coil methods [4] and transmission-line methods [5,6]. Pickup-coil approaches are able to provide reliable low-frequency permeability data, but their operating frequency range is limited by parasitic capacitance and resonant behavior of the coils. Transmission-line approaches are typically more suitable for broadband microwave measurements.

For thin magnetic films, the most commonly used transmission-line approaches are coaxial-line methods [7,8] and planar transmission-line methods [2,3]. Planar fixtures are especially attractive because they are naturally compatible with flat samples and provide direct access of the sample to the microwave-field region, while avoiding the need to fill the entire measurement cross section. This offers an important practical advantage over coaxial-line methods, where films on flexible substrates often have to be rolled into a toroidal form, whereas films on rigid substrates and magnetostrictive films are much less suitable for such preparation. Planar transmission-line methods include both microstrip and coplanar geometries. In the present study, however, we focus on microstrip fixtures, which have been more extensively developed as broadband RF permeameters for quantitative permeability retrieval, whereas coplanar waveguides are more often employed in FMR spectroscopy.

Microstrip permeameters for thin magnetic films have been investigated for more than two decades [5,6,9,10,11,12,13,14,15,16,17,18,19] and remain particularly attractive because they combine relatively high sensitivity to weak magnetic signals with a simple geometry suitable for broadband permeability retrieval. However, despite this long development history, their measurement accuracy remains limited, and their operating frequency range is often constrained by parasitic resonances associated with the fixture and sample dimensions.

There are two principal approaches to improve measurement accuracy and extend the usable frequency range. One is to optimize the fixture geometry and reduce mismatch at the coaxial-to-strip transition [18,19], which is technically demanding. The other is to reduce the electrical size of both the fixture and the sample in order to shift parasitic resonances toward higher frequencies [17], although this approach leads to lower signal-to-noise ratio (SNR). In addition, when the fixture becomes too short, the measurement may become more sensitive to nonuniform fields and to evanescent higher-order modes in the vicinity of the coaxial-to-strip transition.

Another important source of uncertainty arises from the permeability extraction procedure used to obtain permeability from the measured microwave response. In particular, the retrieval accuracy depends not only on the treatment of the effective permeability, but also on the determination of the effective permittivity of the film on substrate and on the subsequent conversion from effective to intrinsic film parameters. These factors show that accurate broadband characterization of thin magnetic films remains sensitive to calibration assumptions, transition nonidealities, fixture resonances, and the conversion from effective to intrinsic material parameters.

In this paper, we develop an improved short-circuited microstrip method for broadband permeability measurements of thin magnetic films. The method is based on a microstrip fixture with a movable shorting wall, which enables full one-port offset-short calibration. This calibration procedure reduces the influence of the non-ideal coaxial-to-strip transition and helps to suppress parasitic resonances associated with the fixture over a broad frequency range, making it possible to use a relatively long fixture and wider samples. In addition, field-dependent normalization to the empty fixture is used to reduce systematic measurement errors associated with the external magnetic field, and a correction for the non-zero effective permeability under a strong external magnetic field is introduced. The method is validated on Co, supermalloy, and FeCo films using independent reference measurements whenever such comparisons are possible, and its performance is demonstrated up to about 20 GHz in the present implementation.

## 2. Materials and Methods

### 2.1. Measurement Fixture and Experimental Setup

**Figure 1:**
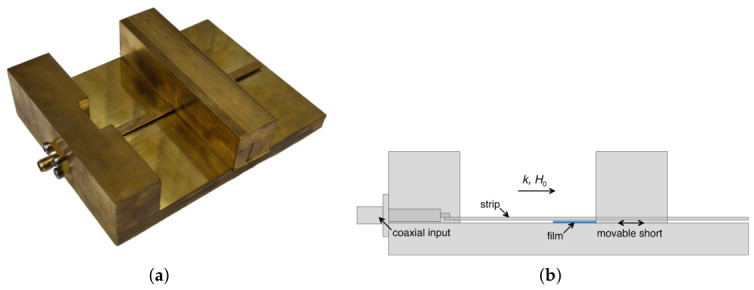
(**a**) Photograph of the short-circuited microstrip fixture. (**b**) Schematic of the measurement geometry.

The measurements were performed using a short-circuited microstrip fixture. A photograph of the fixture is shown in Figure 1. The central strip conductor had a width of 5 mm and a thickness of 1 mm, and the gap between the strip and the ground plane was 1 mm. The movable shorting wall could be moved over a distance of up to 70 mm along the base, providing the offset-short positions required for calibration.

The shorting wall was made of two halves assembled around the central strip conductor. The slot in the wall was machined to match the strip dimensions as closely as possible in order to provide stable and reproducible short-circuit conditions. During measurements, the wall was pressed to the base by a non-magnetic clamp to improve mechanical and electrical contact. Because displacement of the shorting wall may cause mechanical shifts of the strip conductor, the strip was additionally fixed near the coaxial input by attaching it to a dielectric support in the connector region. This was done to improve the mechanical stability of the fixture during repeated calibration and measurement cycles.

The microstrip fixture was connected to a VNA and placed inside an electromagnet capable of generating a uniform external magnetic field of up to 5000 Oe. The static magnetic field was directed along the propagation direction of the microstrip fixture. In the microstrip measurements, the film on PET substrate was placed directly on the ground plane and kept in close contact with the base. During the measurements, the transverse width of the film samples was chosen to be at least three times larger than the strip width in order to cover the main microwave-field region around the strip and reduce edge- related perturbations.

The measurements were interpreted within the quasi-TEM approximation, in which the dominant mode is assumed to remain close to a transverse electromagnetic wave. This approximation is appropriate for thin magnetic films with sufficiently small permeance factor, which is the main case considered in the present work. For thicker samples or films with larger permeance factor, the quasi-TEM approximation becomes less accurate, which may increase the error of permeability retrieval [10,14,15,20].

### 2.2. Calibration Procedure

Calibration is an essential part of broadband microwave measurements in any measurement system. Generally, the relation between the true reflection coefficient $R_{\mathrm{true}}$ of the measured object and the measured reflection coefficient $R_{\mathrm{meas}}$ can be written in the standard form

(1) $$R_{\mathrm{meas}}=E_{\mathrm{DF}}+\frac{E_{\mathrm{RF}}R_{\mathrm{true}}}{1-E_{\mathrm{SF}}R_{\mathrm{true}}},$$

where $E_{\mathrm{DF}}$, $E_{\mathrm{SF}}$ and $E_{\mathrm{RF}}$ are the directivity, source-match, and reflection-tracking error terms, respectively.

In conventional microstrip measurements, full calibration of the fixture is often not readily available. For this reason, in many implementations the calibration is effectively referenced to the coaxial input plane, and the coaxial-to-strip transition is treated as weakly perturbing or nearly ideally matched. Because achieving such matching in practice is technically demanding, several approaches have been proposed to reduce the influence of the coaxial-to-strip transition. One of them is based on approximating the response of the empty fixture by an equivalent-circuit model [11,14]. Calibration procedures using either one [12] or two [21] reference samples have also been proposed. However, the applicability of these approaches is limited by parasitic resonances associated with the fixture length and the dimensions of the reference samples. In addition, the use of reference samples introduces additional uncertainty due to imperfect knowledge of their constitutive parameters and the difficulty of reproducing their exact electromagnetic response in a partially filled microstrip geometry.

As an alternative, the present study employs offset-short calibration [22] implemented by means of a movable shorting wall. For broadband measurements, short standards are especially convenient because, when fabricated with sufficient accuracy, they provide a simple and predictable response over a wide frequency range. Full calibration can therefore be implemented using several controlled short-circuit positions with different offsets. The upper frequency limit of the calibration can be increased by reducing the offset lengths, which shifts the corresponding offset-related resonances to higher frequencies. In addition, the calibration accuracy over different frequency ranges can be improved by combining several offset-short calibrations, i.e., by using more than three offset positions. The main limitation of this approach is the required reproducibility of the empty-cell response for different short positions, after the corresponding phase shift is taken into account.

For an ideal offset short, the reflection coefficient is determined by the phase delay associated with the offset length $l_{i}$, so that

(2) $$R_{i}=-\exp\left(2j\beta l_{i}\right),$$

where $l_{i}$ is the offset of the short standard relative to the zero-phase reference plane, and β is the phase constant of the dominant quasi-TEM mode in the empty air-filled strip section. Since the offset section contains no distributed dielectric substrate, β was approximated by the free-space value $k_{0}$ = 2$\pi /\lambda_{0}$.

The choice of the offset lengths is constrained by two factors. On the one hand, the differences between the offsets must be large enough to provide sufficient phase separation between the calibration standards, especially at the lowest frequencies of interest. On the other hand, at frequencies where the phase differences between two offsets approach integer multiples of π, the calibration becomes poorly conditioned and the uncertainty increases. To reduce this effect over a broad frequency range, two offset-short calibration sets with different offset combinations were used for different frequency sub-bands. The offset set 0, 10 and 20 mm was used in the lower-frequency part of the spectrum, whereas the set 0, 3.5 and 7 mm was used in the higher-frequency part. The two calibrated reflection coefficients were joined by a hard switch at $f_{c}$ = 3 GHz. This crossover frequency was chosen as a compromise between the conditioning of the two calibration sets. At this frequency, the high-frequency offset set provides a phase separation of approximately 25° between the standards, while the low-frequency set remains sufficiently far from its offset-related poorly conditioned region associated with the half-wavelength condition. The continuity of the joining procedure was verified using the validation sample, as described in Section 3.1.

An additional correction was introduced by normalizing the measured response to that of the empty fixture at each value of the external magnetic field. This step was motivated by the experimentally observed field dependence of the empty-cell response. In practice, this normalization substantially reduces field-dependent systematic distortions of the transfer response of the measurement tract. Accordingly, the complete calibration procedure used in this work consisted of direct offset-short calibration of the microstrip fixture followed by field-dependent normalization to the empty-cell measurement.

### 2.3. Transmission-Line Model and Determination of Effective Parameters

To describe the sample-loaded microstrip fixture, a transmission-line approach was used. Within this model, the section containing the sample is described in terms of the effective permeability $\mu_{\mathrm{eff}}$ and effective permittivity $\epsilon_{\mathrm{eff}}$. The normalized input impedance of a short-circuited loaded section is written as

(3) $$Z_{\mathrm{in}}=\sqrt{\frac{\mu_{\mathrm{eff}}}{\varepsilon_{\mathrm{eff}}}}\tanh\left(j\frac{2\pi L_{s}}{\lambda }\sqrt{\varepsilon_{\mathrm{eff}}\mu_{\mathrm{eff}}}\right),$$

where λ is the free-space wavelength and $L_{s}$ is the sample length along the propagation direction. The corresponding reflection coefficient is then given by

(4) $$R=\frac{Z_{\mathrm{in}}-1}{Z_{\mathrm{in}}+1},$$

In the first step of the retrieval procedure, the effective permittivity $\epsilon_{\mathrm{eff}}\left(f\right)$ of the sample-loaded fixture is determined from the measurement performed in a sufficiently strong external magnetic field. In this state, the magnetic response of the film is strongly suppressed, so that the saturated-state measurement can be used as the basis for permittivity retrieval. The residual deviation of the effective permeability from unity in the saturated state is treated separately, as described in Section 2.5. After $\epsilon_{\mathrm{eff}}\left(f\right)$ has been determined, the permeability spectrum is retrieved from the zero-field measurement of the same sample-loaded fixture. This retrieval procedure is also applicable to measurements performed at nonzero external magnetic fields, with the corresponding field-dependent permeability spectrum obtained in the same manner.

For thin magnetic films, accurate determination of $\epsilon_{\mathrm{eff}}$ is particularly important because the thickness of the magnetic film is typically much smaller than that of the substrate. As a result, even a relatively small error in the retrieved effective permittivity may lead to a substantial error in the subsequent reconstruction of the film permeability, especially at high frequencies. For this reason, it is preferable to determine $\epsilon_{\mathrm{eff}}$ directly from measurement of the film on substrate under a strong external magnetic field, rather than from a separate substrate-only measurement. Using a substrate without the film may introduce additional uncertainty due to imperfect reproducibility of substrate dimensions, positioning, and loading conditions inside the fixture.

Because the films are in-plane anisotropic, the scalar quantity $\mu_{\mathrm{eff}}$ used in the retrieval should be understood as the effective permeability component associated with the direction of the microwave magnetic field, rather than as an isotropic material permeability. In the present measurements, the films were oriented so that the microwave magnetic field was directed along the hard axis, where a strong transverse magnetic response is expected. The same fixture geometry, however, can also be used with a different in-plane orientation of the film to probe another permeability component. Possible tensor and gyrotropic effects are not separately resolved in the present one-port scalar retrieval and are included only through this effective measured component.

### 2.4. Conversion from Effective to Intrinsic Permeability

After the effective parameters of the sample-loaded microstrip section have been retrieved, an additional step is required to convert them into the intrinsic permeability of the magnetic film. Among rigorous quasi-static approaches used for this purpose, the most common are the variational method [9,23] and conformal-mapping techniques [13,24]. In the present study, the variational method [25] was used because it can be adapted to different fixture geometries and sample-filling configurations through an appropriate choice of Green’s function.

Along with such rigorous approaches, a simpler approximate treatment is also widely used [4,5,6,11]. In this case, the conversion from the effective permeability to the intrinsic permeability is performed using a simple empirical scaling relation

(5) $$\mu_{\mathrm{int}}=\frac{K}{t}\left(\mu_{\mathrm{eff}}-1\right)+1,$$

where $\mu_{\mathrm{int}}$ is the intrinsic permeability of the film, *t* is the film thickness, and *K* is an effective geometric filling coefficient. This relation is convenient because it reduces the conversion problem to a single empirical parameter, which may be estimated experimentally using a reference sample. However, several studies have pointed out that the use of a constant *K* may introduce significant error [14,15].

**Figure 2:**
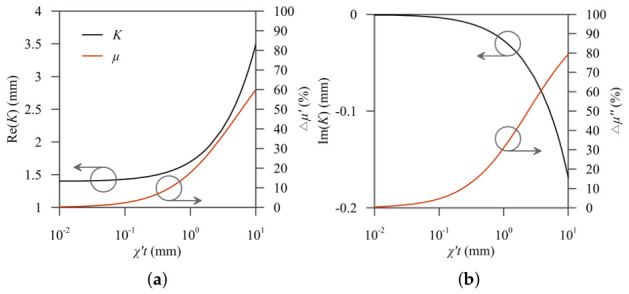
Dependence of the real (**a**) and imaginary (**b**) parts of the geometric coefficient *K* on the permeance factor $\chi^{\prime }t$ for the present microstrip geometry, and the corresponding relative error in intrinsic permeability retrieval when a constant value K=1.4 mm is used.

In the present work, the behavior of the coefficient *K* for the microstrip geometry under study was evaluated using the variational method. Introducing the magnetic susceptibility χ=μ−1, the permeance factor is defined as $\chi^{\prime }t$ and characterizes the magnetic loading of the line. As shown in Figure 2, *K* is not constant but depends on the permeance factor of the film. Figure 2 also shows the relative error in intrinsic permeability retrieval when the low-permeance limiting value of *K* is used over the entire range. For the present fixture, this limiting value is approximately K≈1.4 mm. The calculation shown in Figure 2 was performed for a magnetic loss tangent of 0.1; for other loss tangents, the order of magnitude of the error estimate remains similar.

For films with permeance factor $\chi^{\prime }t$ below approximately 0.1 mm, the error introduced by using the low-permeance limiting value of *K* does not exceed about 10% for the present geometry. At larger permeance factors, however, the error may become substantial. Therefore, in the present work the simple empirical scaling relation was used only to illustrate the limits of this approximation, whereas the actual permeability retrieval was performed using the full variational calculation.

### 2.5. Correction for Residual Permeability in the Saturated State

As described in Section 2.3, in the present work the permittivity spectrum $\epsilon_{\mathrm{eff}}\left(f\right)$ is retrieved from measurement of the film on substrate under a strong external magnetic field $H_{\mathrm{sat}}$. This procedure implicitly assumes that $\mu_{\mathrm{eff}}\left(H_{\mathrm{sat}}\right)=1$. However, even under a strong external magnetic field $H_{\mathrm{sat}}$, the film retains a finite static susceptibility. As a result, a part of the magnetic contribution present in the measurement at $H_{\mathrm{sat}}$ is incorporated into the retrieved effective permittivity, which subsequently leads to an underestimation of the permeability spectrum reconstructed at zero field.

Within the uniaxial approximation, the static susceptibility in an external field *H* can be written as

(6) $$\chi \left(H\right)=\frac{4\pi M_{s}}{H_{k}+H},$$

where $M_{s}$ is the saturation magnetization and $H_{k}$ is the effective anisotropy field.

Even under a strong external magnetic field $H_{\mathrm{sat}}$, the residual susceptibility remains finite. In the thin-film limit, neglecting this contribution leads, to the first approximation, to

(7) $$\chi_{0}^{\mathrm{app}}\approx \chi_{0}-\chi_{\mathrm{sat}},$$

where $\chi_{0}$ is the susceptibility at zero field and $\chi_{\mathrm{sat}}$ is the susceptibility at field $H_{\mathrm{sat}}$. Hence, the relative error in the recovered static susceptibility can be estimated as

(8) $$\delta_{x}=\frac{\chi_{0}-\chi_{0}^{\mathrm{app}}}{\chi_{0}}\approx \frac{\chi_{\mathrm{sat}}}{\chi_{0}}=\frac{H_{k}}{H_{k}+H_{\mathrm{sat}}}.$$

This shows that the error becomes appreciable when $H_{k}$ is not small compared with $H_{\mathrm{sat}}$, whereas for films with smaller $H_{k}$ the same residual contribution under the field $H_{\mathrm{sat}}$ corresponds to a smaller relative error.

The same effect can also be identified from the behavior of the retrieved effective permittivity under the field $H_{\mathrm{sat}}$. In the low-frequency limit, the input impedance of the short-circuited loaded section is more sensitive to μ than to ϵ, which results in a nonphysical low-frequency upturn of the retrieved $\epsilon_{\mathrm{eff}}$. In the first approximation, if $\mu_{\mathrm{eff}}\left(H_{\mathrm{sat}}\right)=1+\Delta \mu_{\mathrm{eff}}$, this distortion can be described as

(9) $$\varepsilon_{\mathrm{eff}}^{\mathrm{app}}\left(f\right)\approx \varepsilon_{\mathrm{eff}}^{\mathrm{true}}\left(f\right)+\frac{C\Delta \mu_{\mathrm{eff}}}{f^{2}}.$$

where *C* is a geometry-dependent coefficient. A $1/f^{2}$-type low-frequency rise of $\epsilon_{\mathrm{eff}}$ can be regarded as an indication that the residual permeability under the field $H_{\mathrm{sat}}$ has not been taken into account properly. In the present work, the residual permeability correction is determined directly from the spectrum under the field $H_{\mathrm{sat}}$. The optimal value of $\mu_{\mathrm{eff}}\left(H_{\mathrm{sat}}\right)=1+\Delta \mu_{\mathrm{eff}}$ is found by optimizing the reconstructed permittivity spectrum $\epsilon_{\mathrm{eff}}\left(f\right)$ so that its frequency dispersion is minimized. This correction should be understood as a first-order correction for the residual low-frequency susceptibility under the field $H_{\mathrm{sat}}$, rather than as a reconstruction of the full frequency-dependent permeability $\mu_{\mathrm{eff}}\left(H_{\mathrm{sat}},f\right)$. Real dielectric dispersion of the film/substrate/fixture system remains included in the reconstructed $\epsilon_{\mathrm{eff}}\left(f\right)$, whereas the correction suppresses only the specific $1/f^{2}$-type low-frequency rise caused by residual permeability being incorrectly attributed to effective permittivity. A possible dynamic magnetic contribution under $H_{\mathrm{sat}}$ may still occur at higher frequencies, where the FMR response is shifted by the external field, but its influence can be further minimized by increasing the saturating field.

### 2.6. Samples and Reference Measurements

Two independent reference approaches were used in this work when applicable. The first was the coaxial transmission-line method [8,26], which provided broadband reference spectra for samples that could be prepared in coaxial form without significant magnetostriction-induced distortion. In the coaxial reference experiment, the film was cut into long strips with a total length of about 1.5 m and rolled into a dense washer to fill the cross section of a 7/3 mm coaxial line. To account for possible magnetostriction effects during rolling, two configurations were measured, corresponding to the film surface facing inward and outward.

The second reference approach was reconstruction of the FMR-related contribution to the permeability spectrum from static magnetic measurements using hysteresis loops measured along the hard axis [27]. For this purpose, the imaginary part of the permeability was estimated using an approach based on the Barandiaran method [28]. First, the distribution of anisotropy fields was derived from the hard-axis hysteresis loop. This distribution was then converted into a distribution of resonance frequencies using the Kittel relation. Assuming a set of independent high-Q resonances, the resulting resonance-frequency distribution was used to calculate $\mu^{\prime \prime }\left(f\right)$, and the corresponding $\mu^{\prime }\left(f\right)$ spectrum was then reconstructed using a Lorentzian representation of the resonances [29].

All films studied in this work were deposited by magnetron sputtering on 12 μm thick flexible polyethylene terephthalate (PET) substrates. During measurements in the short-circuited microstrip fixture, the microwave magnetic field was oriented along the hard axis of the films. The lengths of the supermalloy and FeCo film samples were 5 mm, whereas the corresponding length of the Co sample was 40 mm in order to increase the signal level.

Three samples with substantially different thicknesses and magnetic properties were selected to evaluate the method under complementary conditions. The first was a 17 nm Co film, used primarily to assess the lower sensitivity limit of the method for ultrathin magnetic films. Because rolled specimens for coaxial measurements were strongly affected by magnetostriction, this sample could not be reliably characterized by the coaxial reference method and was therefore compared only with permeability spectra reconstructed from static magnetic measurements.

The second sample was a 160 nm supermalloy film of composition ${\mathrm{Ni}}_{79}{\mathrm{Fe}}_{17}{\mathrm{Mo}}_{4}$ [30]. Owing to its weak magnetostriction, this sample could be compared with both independent reference approaches and therefore served as the primary validation sample for the proposed method.

The third sample was a 1.2 μm FeCo film. This sample was used to examine the applicability of the method to films with a broader permeability spectrum and to evaluate the correction for residual permeability under the field $H_{\mathrm{sat}}$. Owing to its weak magnetostriction, the FeCo film could also be measured by the coaxial method. However, comparison with permeability spectra reconstructed from static magnetic measurements was not considered reliable for this sample because of its more complex magnetic structure.

## 3. Results and Discussion

### 3.1. Validation of the Proposed Method

**Figure 3:**
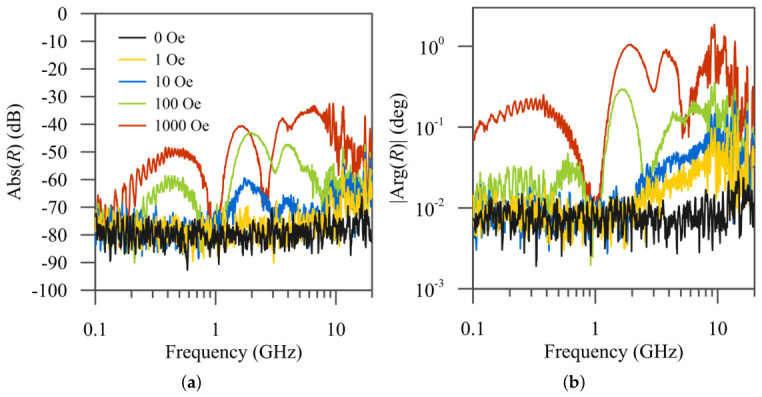
The magnitude (**a**) and phase (**b**) of the reflection coefficient for the empty fixture measured at different DC magnetic fields.

In the present work, the permeability spectra were retrieved using a combination of full one-port offset-short calibration and field-dependent empty-cell normalization. The latter correction was introduced because the measured response of the empty fixture was found to depend on the applied external magnetic field. As shown in Figure 3, the reflection coefficient of the empty cell changes noticeably with field over a wide frequency range, indicating that the measurement tract itself is affected by the DC magnetic field. For this reason, empty-cell normalization at each field value was included in all subsequent retrieval procedures. For clarity of presentation, the spectra obtained from the microstrip measurements were smoothed using a five-point moving-average filter. This smoothing was applied only to reduce random point-to-point noise and did not affect the retrieval procedure.

The origin of the field-dependent empty-cell response was additionally examined by modifying the input connection of the fixture. The dominant contribution was found to be associated with the coaxial connector/cable junction rather than with the sample region of the fixture. In particular, when the connector–cable junction was replaced by a rigid soldered electrically continuous connection, the field dependence of the empty-cell response was strongly suppressed. This indicates that the observed effect is mainly caused by field-sensitive contact or transition conditions in the input part of the measurement tract. Since this field-sensitive section is located before the sample-loaded region, its contribution is expected to be reproducible for the empty and sample-loaded measurements and can therefore be reduced by normalization to the empty fixture measured at the same external field.

**Figure 4:**
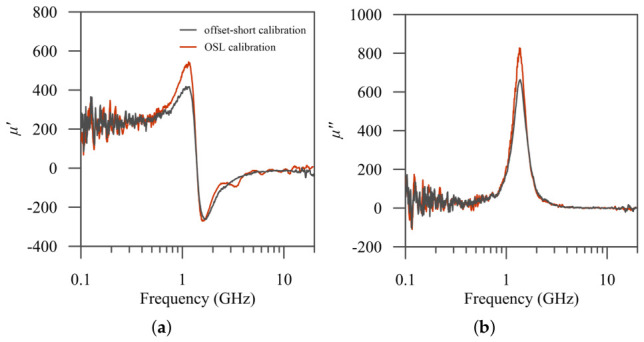
The frequency dependencies of the real (**a**) and imaginary (**b**) parts of the complex permeability for the 160 nm supermalloy film retrieved using offset-short and OSL calibration procedures.

After introducing field-dependent normalization, the influence of the calibration procedure itself was evaluated using the 160 nm supermalloy film as a reference sample. Figure 4 compares the permeability spectra retrieved from the same measurement data using two calibration approaches: conventional OSL calibration performed at the coaxial input plane and the proposed offset-short calibration implemented directly with the microstrip fixture.

The comparison shows that the offset-short calibration procedure more effectively suppresses parasitic resonances associated with the electrical length of the fixture and also provides a more accurate response amplitude in the resonance region.

Since the offset-short calibration was assembled from two sub-band calibrations, the continuity at the crossover frequency was additionally checked using the 160 nm supermalloy film. Both calibration sets were applied independently to the same measurement data in the vicinity of $f_{c}$ = 3 GHz, and the resulting permeability spectra were compared. The spectra obtained with the two calibrations were visually indistinguishable on the scale of the main plots. Therefore, the comparison is shown in terms of the relative difference

(10) $$\Delta \mu =100\left|\frac{\mu_{\mathrm{LF}}-\mu_{\mathrm{HF}}}{\mu_{\mathrm{HF}}}\right|.$$

**Figure 5:**
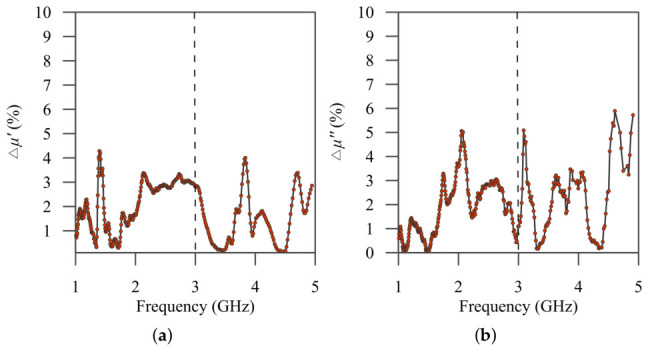
The relative difference between the permeability spectra retrieved from the same measurement data using the low-frequency calibration set 0, 10 and 20 mm and the high-frequency calibration set 0, 3.5 and 7 mm: (**a**) $\Delta \mu^{\prime }$ and (**b**) $\Delta \mu^{\prime \prime }$. The vertical dashed line indicates the crossover frequency $f_{c}$ = 3 GHz used for the hard switch between the two calibrations.

As shown in Figure 5, in the range 1–3 GHz the mean relative difference was about 2%, with local deviations not exceeding approximately 5%. No step-like feature is observed at the crossover frequency, indicating that the sub-band joining does not introduce an artificial kink into the retrieved permeability spectrum.

**Figure 6:**
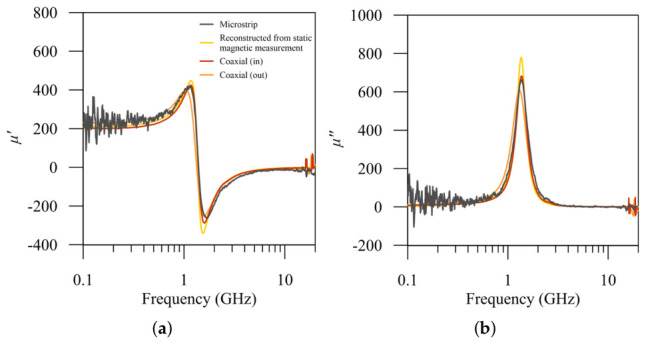
The frequency dependencies of the real (**a**) and imaginary (**b**) parts of the complex permeability for the 160 nm supermalloy film measured by the short-circuited microstrip method and compared with the spectra obtained by the coaxial method and by reconstruction from static magnetic measurement.

The resulting spectra were then compared with independent reference measurements to evaluate whether the proposed offset-short calibration yields a more physically reliable permeability spectrum. Figure 6 compares the permeability spectrum measured by the short-circuited microstrip method with the spectra obtained by the coaxial and static reference approaches. Good agreement is observed in the overall spectral shape, the resonance position, and the amplitude level of both the real and imaginary parts of the permeability.

### 3.2. Sensitivity Limit

**Figure 7:**
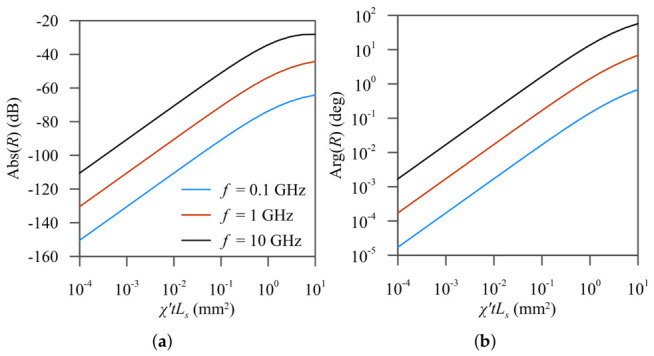
Calculated magnitude (**a**) and phase (**b**) of the reflection-coefficient response for magnetic films as a function of magnetic conductance $\chi^{\prime }tL_{s}$ at 0.1, 1 and 10 GHz.

After validating the proposed method, it is useful to estimate the practical sensitivity limit in more general terms. For this purpose, the expected signal from magnetic films was evaluated using the variational approach together with Equations (3) and (4). Figure 7 shows the calculated response of magnetic samples characterized by the magnetic conductance $\chi^{\prime }tL_{s}$, where t and $L_{s}$ are the film thickness and length along the propagation direction, respectively. In these calculations, the magnetic loss tangent was taken to be 0.1; larger losses would lead to a higher signal level. The calculated dependencies show that the response increases with frequency and with magnetic conductance, which is consistent with the experimentally observed behavior of the measured films. For similar fixture geometries, the sensitivity is governed mainly by the gap size and is, to first approximation, inversely proportional to the gap.

The calculated curves in Figure 7 also indicate that the phase of the reflection coefficient is more sensitive to weak magnetic loading than the magnitude. This trend is consistent with the experimental background measurements shown in Figure 3, where the empty-cell response at zero field remains at a level of about −80 dB in magnitude and about $10^{-2}$ deg in phase over a substantial part of the frequency range. Comparison of this background with the calculated signal levels makes it possible to estimate the practical sensitivity limit of the present implementation. In terms of magnetic conductance, the experimentally accessible range corresponds approximately to values above 1 ${\mathrm{mm}}^{2}$ at 0.1 GHz, above 0.1 ${\mathrm{mm}}^{2}$ at 1 GHz, and above 0.01 ${\mathrm{mm}}^{2}$ at 10 GHz.

The usable frequency range of the method is limited by different mechanisms at the low- and high-frequency ends. At low frequencies, the main limitation is the random background noise of the measurement system, since the magnetic response of thin films decreases with decreasing frequency. At high frequencies, the signal-to-noise ratio is not the dominant limitation because the magnetic response generally increases with frequency. Instead, the upper part of the band is mainly limited by calibration stability and mechanical reproducibility of the fixture. In particular, small shifts of the strip conductor during movement of the shorting wall may slightly modify the effective response of the non-ideal coaxial-to-strip transition region, leading to increased systematic uncertainty. The offset-short calibration also becomes more sensitive to small mechanical and phase errors at higher frequencies. Accordingly, the upper limit of about 20 GHz should be understood as a practical limit of the present fixture and calibration implementation, where systematic uncertainty gradually increases, rather than as a sharp physical cutoff.

### 3.3. Sensitivity Demonstrated on an Ultrathin Co Film

**Figure 8:**
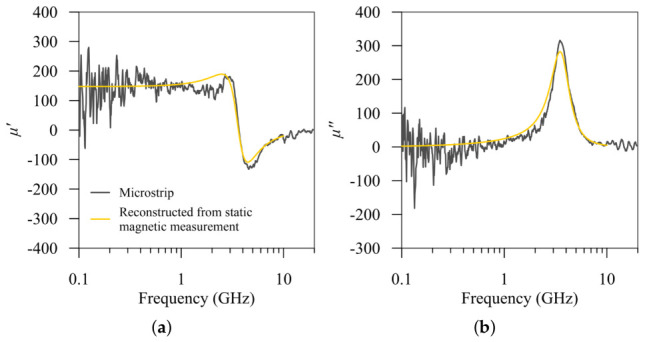
The frequency dependencies of the real (**a**) and imaginary (**b**) parts of the complex permeability of the 17 nm Co film measured by the short-circuited microstrip method and compared with the spectrum reconstructed from static magnetic measurement.

To assess the lower practical sensitivity limit of the proposed method, an ultrathin Co film with a thickness of 17 nm was measured. Figure 8 compares the permeability spectrum measured in the short-circuited microstrip fixture with the spectrum reconstructed from the hysteresis loop. Despite the noticeably higher noise level, the measured spectrum is in good agreement with the reference reconstruction from static magnetic measurements.

**Figure 9:**
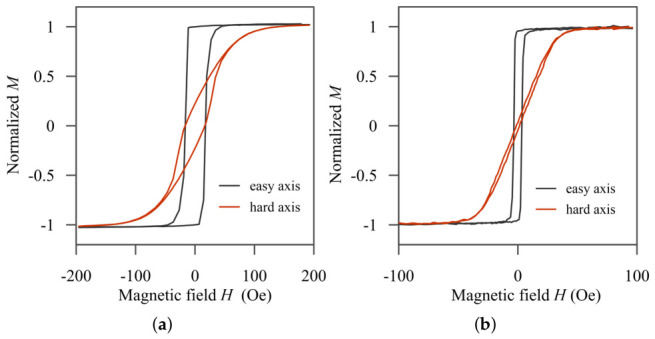
Hysteresis loops of the 17 nm Co film (**a**) and the 160 nm supermalloy film (**b**) measured along the hard and easy axes.

The hysteresis loops used for the reconstruction of the Co and supermalloy spectra are shown in Figure 9. The loops measured along the easy and hard axes confirm clear in-plane uniaxial anisotropy in both samples. The effective anisotropy fields were estimated from the static anisotropy energy, calculated from the difference between the integrals of the easy- and hard-axis hysteresis loops, following the standard loop-area approach [31]. The resulting values are approximately $H_{k}\approx 70\;\mathrm{Oe}$ for Co and $H_{k}\approx 65\;\mathrm{Oe}$ for the supermalloy film.

### 3.4. Effect of Residual-Permeability Correction on the FeCo Film

To evaluate the effect of correcting residual saturated permeability under conditions where this contribution is expected to be non-negligible, a 1.2 μm thick FeCo film was measured. This sample is characterized by a relatively broad permeability spectrum and a comparatively large effective anisotropy field $H_{k}\approx 700\;\mathrm{Oe}$. For this reason, neglecting the residual permeability in the saturated state produces a more pronounced error than in softer films with smaller $H_{k}$.

**Figure 10:**
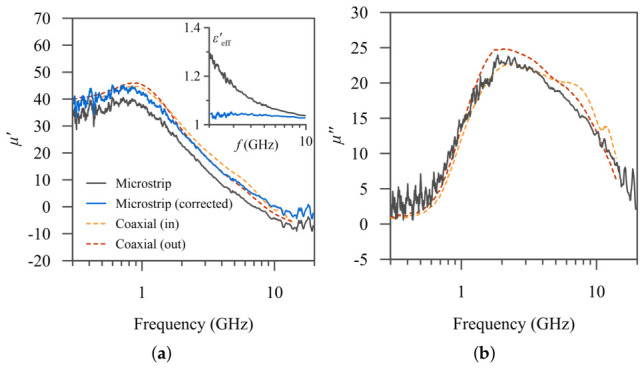
The frequency dependencies of the real (**a**) and imaginary (**b**) parts of the complex permeability of the 1.2 μm FeCo film measured by the short-circuited microstrip method before and after correction for residual saturated permeability, compared with coaxial reference data. The inset shows the retrieved effective permittivity spectra with and without the $\mu_{\mathrm{eff}}\left(H_{\mathrm{sat}}\right)$ correction.

Figure 10 compares the permeability spectrum retrieved from the short-circuited microstrip measurement before and after correction for residual $\mu_{\mathrm{eff}}\left(H_{\mathrm{sat}}\right)$ together with the spectra obtained by the coaxial reference method. Without this correction, the microstrip result exhibits a systematic underestimation of the real part of the permeability and a noticeable shift of the spectral curve. The difference in the amplitude level of $\mu^{\prime }$ spectra before and after correction is about 5, which corresponds to a relative change exceeding 10%. The inset in Figure 10 shows the corresponding retrieved $\epsilon_{\mathrm{eff}}$ spectra. Before correction, the effective permittivity exhibits a pronounced low-frequency upturn, whereas after correction this nonphysical behavior is strongly reduced. After correction, the microstrip spectrum becomes noticeably closer to the coaxial reference measurement.

## 4. Conclusions

An improved short-circuited microstrip method for broadband measurement of the complex permeability of thin magnetic films was presented in this paper. This technique does not require known reference samples or strict electromagnetic optimization of the coaxial-to-strip transition to minimize its impact on the measurement. The method combines full one-port offset-short calibration implemented directly in the microstrip fixture, field-dependent normalization to the empty fixture, and correction for residual permeability under a strong external magnetic field. These steps reduce systematic errors associated with the non-ideal coaxial-to-strip transition, field-dependent fixture response, and incomplete suppression of the magnetic contribution at saturation field. The operating frequency range extends to 20 GHz with gradually increasing uncertainty rather than a sharp cutoff due to calibration stability and mechanical reproducibility. Future work may focus on reducing the field sensitivity of the fixture and on improving mechanical reproducibility of the movable shorting wall.

## Data Availability

The original contributions presented in this study are included in the article. Further inquiries can be directed to the corresponding author.

## Abbreviations

The following abbreviations are used in this manuscript:

RF	Radio frequency
FMR	Ferromagnetic resonance
VNA	Vector network analyzer
OSL	Open-Short-Load
PET	Polyethylene terephthalate
TEM	Transverse electromagnetic
VSM	Vibrating-sample magnetometer
DC	Direct current
SNR	Signal-to-noise ratio
